# The Role of Adaptation in Bacterial Speed Races

**DOI:** 10.1371/journal.pcbi.1004974

**Published:** 2016-06-03

**Authors:** Jérôme Wong-Ng, Anna Melbinger, Antonio Celani, Massimo Vergassola

**Affiliations:** 1 University of California San Diego, Department of Physics, La Jolla, California, United States of America; 2 The Abdus Salam International Centre for Theoretical Physics (ICTP), Trieste, Italy; University of Illinois at Urbana-Champaign, UNITED STATES

## Abstract

Evolution of biological sensory systems is driven by the need for efficient responses to environmental stimuli. A paradigm among prokaryotes is the chemotaxis system, which allows bacteria to navigate gradients of chemoattractants by biasing their run-and-tumble motion. A notable feature of chemotaxis is adaptation: after the application of a step stimulus, the bacterial running time relaxes to its pre-stimulus level. The response to the amino acid aspartate is precisely adapted whilst the response to serine is not, in spite of the same pathway processing the signals preferentially sensed by the two receptors Tar and Tsr, respectively. While the chemotaxis pathway in *E. coli* is well characterized, the role of adaptation, its functional significance and the ecological conditions where chemotaxis is selected, are largely unknown. Here, we investigate the role of adaptation in the climbing of gradients by *E. coli*. We first present theoretical arguments that highlight the mechanisms that control the efficiency of the chemotactic up-gradient motion. We discuss then the limitations of linear response theory, which motivate our subsequent experimental investigation of *E. coli* speed races in gradients of aspartate, serine and combinations thereof. By using microfluidic techniques, we engineer controlled gradients and demonstrate that bacterial fronts progress faster in equal-magnitude gradients of serine than aspartate. The effect is observed over an extended range of concentrations and is not due to differences in swimming velocities. We then show that adding a constant background of serine to gradients of aspartate breaks the adaptation to aspartate, which results in a sped-up progression of the fronts and directly illustrate the role of adaptation in chemotactic gradient-climbing.

## Author Summary

Biological sensory pathways are presumed to evolve for the processing of environmental information, yet quantitative evidence is scant. Chemotaxis allows bacteria to sense chemical gradients but their ecological distribution, e.g. whether natural gradients sensed by *E. coli* change slowly or rapidly in space and time, is unknown. That distribution matters, as it controls constraints and selective pressure acting on the pathway. We used microfluidic devices to generate controlled chemoattractant gradients and measure the speed of bacterial climbing of those gradients. We could thereby assay the impact of adaptation properties of the chemotaxis pathway onto the progression of gradient climbing. We specifically show that loss of adaptation, induced by adding a background of serine to gradients of aspartate, leads to a faster progression of the bacteria along the chemoattractant gradient. We finally discuss why our experiments suggest that ecological conditions are likely to involve chemoattractant profiles more complex than constant gradients usually considered in the laboratory.

## Introduction

A major example of adaptation in transduction pathways is bacterial chemotaxis, where saturating stimuli are followed by a precise recovery of the cell’s pre-stimulus tumbling frequency [1, 2]. The recovery is achieved by an integral feedback control [3], which is molecularly mediated by the methylation of chemoreceptor clusters [4–7]. More recently, the output of the chemotaxis pathway was also found to adapt via motor remodeling [8]. Time scales of adaptation are on the order of ten minutes for 1mM of L-aspartate and shorten as the amplitude of the stimulus reduces [9, 10]. In the linear regime, adaptation takes place over a few seconds, as illustrated by the chemotactic impulse responses measured either by tethering [11] or by trajectories of free bacteria [12].

The *E. coli* chemotaxis pathway has two major amplification steps: the first is at the level of the clusters of receptors localized at the cell poles [13–16]; the second is at the level of the motors controlling the rotation of flagella [17]. Their combination results in gains of several hundreds that allow the detection of tiny variations in the concentration of chemicals [5]. Maintaining that benefit over an extended dynamic range of concentrations is the oft-heard justification for adaptation.

Adaptation is not precise for all attractants and concentrations, though. A well-known example is the chemoattractant serine, preferentially bound by the Tsr receptor. In standard conditions of bacterial preparation and culture, the frequency of tumbling reduces as the concentration of serine increases [18]. Loss of precise adaptation is due to the reduced availability of occupation sites for (de-)methylation on the receptor clusters [4–7]. In the linear regime, adaptation is quantified by the integral of the impulse response and precise adaptation corresponds to a vanishing integral [11]. Both serine and aspartate feature a two-lobe response, yet for serine the areas of the positive and the negative lobes differ [12]. Lack of adaptation is also observed for the chemorepulsion to leucine [12]. Even for aspartate (or its non-metabolizable analogue alpha-methyl-DL-aspartate), the *E. coli* chemotaxis pathway shows imprecise adaptation at high concentrations [19, 20].

Is the lack of precise adaptation an imperfection (as its common designation “imperfect adaptation” tends to suggest) or does it actually have functional relevance? No major impairment of motility is observed for chemotaxis in serine [18, 21–24]. Furthermore, theoretical arguments suggest that lack of precise adaptation might actually bring some advantages. First, the chemotactic velocity in a static constant gradient is predicted to be larger if the chemotaxis response is not adapted [25]. Second, accumulation at peaks of concentration should be favored by breaking adaptation [26]. Finally, the optimal degree of adaptation should depend on the spatial and temporal profile of the chemoattractant fields [27].

Previous theoretical works all employ linear response theory, which disregards the variations of sensitivity, dynamic range and response as bacteria progress along the gradients. These effects are crucial as variations might reduce the bacterial response and overwhelm the aforementioned effects, which assume that the response remains fixed. In particular, this limitation makes the prediction [25] of a stronger chemotactic velocity in the absence of perfect adaptation not conclusive, and the issue remains moot.

Theoretical issues are reviewed and discussed in the first section below, which provides the motivation for the experiments that are reported in the rest of the paper. In order to conclusively resolve the issue, we developed microfluidic techniques and built a setup where bacteria climb two static concentration gradients of aspartate and serine, otherwise identical in shape and intensity. The rationale for our set-up is that bacteria come from the same population so as to reduce variability due to different cultures and/or conditions. Gradients of chemoattractants span an extended range of concentration, from micromolar values at the entry of the channel to mM’s in the reservoirs used to establish the gradients. We also engineered gradients of aspartate with a uniform background of serine. The integration of the two signals leads to loss of precise adaptation to aspartate, which can be used to directly assess the role of adaptation in the progression of bacteria along the gradients. We quantified the progression by measuring the cumulative distribution of bacteria into the channels and by tracking the position of the most advanced bacteria (the 10^th^, 20^th^ and 40^th^) as a function of time. We could thereby compare the progression of the bacterial fronts for different chemoattractants and conditions, as we report below.

## Results

### Why loss of precise adaptation could be advantageous to increase the chemotactic velocity

Linear response theory cannot be employed to conclusively analyze the progression of bacteria over an extended range of concentrations. Indeed, the amplitude and the form of the linear response kernel (the function *K*(*t*) defined below) change as bacteria climb the gradients. Furthermore, the state around which one should linearize is generally different from the resting state [28] and systematically drifts if adaptation is not precise. The scope of this section is to present a qualitative analysis based on linear response theory, which highlights the mechanisms involved in the dynamics and the uncertainties on quantitative numerical factors that are crucial for a conclusive answer. This sets the stage for the experiments presented in the following sections.

In the linear response regime, the chemotactic velocity along the concentration gradient is the product of the gradient and the chemotactic coefficient *χ*, which has the expression [27]:

$$\chi =\frac{\alpha u^{2}}{3\sigma^{2}}\times \int_{0}^{\infty }e^{-\sigma t}K\left(t\right)dt\;.$$ (1)

Here, the impulse response *K*(*t*) is the change in the probability of running (vs tumbling) at time *t* as a unit impulse in concentration is imparted at time 0. The time-integral $\int_{0}^{\infty }K\left(t\right)dt$ measures the derivative of the steady-state probability of running with respect to the concentration, i.e. a zero value corresponds to precise adaptation.

The other parameters in Eq (1)

$$\alpha =\frac{1-\langle \cos\varphi \rangle }{\tau_{r}}\;,\;\sigma =2D_{rot}+\alpha \;,$$ (2)

involve the angle *φ* of scatter during tumblings (the experimental value is 〈cos *φ*〉 ≃ 0.3) and the rotational diffusivity is approximately *D*_*rot*_ ≃ 0.1 rad^2^/s in standard laboratory conditions [5]. Note that Eq (1) differs from estimates based on a single run, e.g. those used in [25, 28]. In particular, the proper limit to times longer than the microscopic decorrelation times is taken, which ensures that time-correlations among successive runs are well captured [27]. Note also that, at variance with the prior prediction in Ref. [29], the expression (1) correctly captures the chemotactic velocity when adaptation is not precise (see SM for a comparison with numerical simulations).

As we mentioned above, the expression (1) has the limitation that it should only be understood as referring to local values. For instance, in the absence of adaptation, the running time *τ*_*r*_ varies with the position along the gradient whilst the running speed *u* ≃ 15*μ*m/s is roughly constant in our conditions. The form and the amplitude of the linear response kernel (see Eq (3)) are expected to vary along the gradient. Their dependence on the position along the gradient constitutes the main factor of uncertainty as it directly controls the local value of the up-gradient velocity (see, e.g., Eq (4)).

The first term in Eq (1) is proportional to *u*^2^ *τ*_*r*_ for *D*_*rot*_ *τ*_*r*_ small and to $u^{2}/(D_{rot}^{2}\tau_{r})$ in the opposite limit, i.e. it increases with *τ*_*r*_ if the running time is smaller than the correlation time set by the rotational diffusivity. The first behavior *u*^2^ *τ*_*r*_ is the only possible dimensional combination when *D*_*rot*_ is neglected. The product *u* × *uτ*_*r*_∇*c* is intuited as the velocity times the difference in concentration across a run, which is the signal driving the bias of the run along the concentration gradient. Conversely, extending runs beyond ∼1/*D*_*rot*_ is not efficient. Indeed, trajectories of duration *τ*_*r*_ are then roughly composed of *n* = *τ*_*r*_ *D*_*rot*_ stretches of length *u*/*D*_*rot*_ and independent orientation. Only one of those *n* stretches is biased, though, so that the behavior above *u*^2^/*D*_*rot*_ × 1/*n* is obtained. Note that *D*_*rot*_ *τ*_*r*_ is small for *E. coli* (see data in the sequel).

The second integral term in Eq (1) is conveniently recast using specific expressions for experimental responses [11, 12]:

$$K\left(t\right)=K_{0}\lambda e^{-\lambda t}\left[\lambda t-\frac{\left(1-A\right)}{2}{\left(\lambda t\right)}^{2}\right]\;.$$ (3)

Here, *A* controls the loss of precise adaptation, the timescale *λ*^−1^ controls the memory of the past concentration detections and the amplitude *K*_0_ reflects the sensitivity and the dynamic range of the response. Experiments for serine give *A* ≃ 0.03 in the range 5*μ*M to 500*μ*M and *λ* ≃ 1*s*^−1^ with a weak dependence on concentration [12]. The response to aspartate is also well described by Eq (3) with *A* = 0 (precise adaptation). Elementary integrals in Eq (1) for the form Eq (3) yield:

$$\chi =K_{0}\left[\frac{u^{2}\alpha }{3\sigma^{2}}\times \frac{\lambda^{2}\left(\sigma +A\lambda \right)}{{\left(\sigma +\lambda \right)}^{3}}\right]\;.$$ (4)

**Fig 1:**
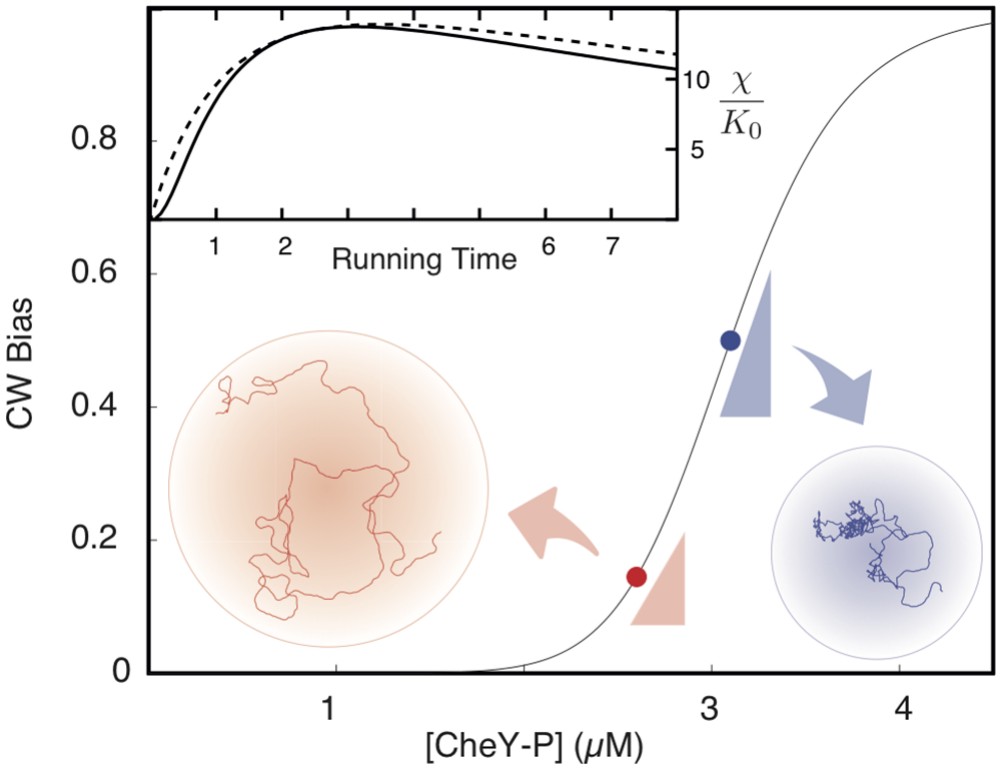
A sketch of the motor response curve and the dependence of the up-gradient chemotactic velocity on the running time. The curve represented in the main panel is a cartoon version of the clockwise (CW) bias *vs* the concentration of the second messenger CheYp of the chemotaxis pathway. Having the system set at the inflection point of the curve (blue point) would maximize the slope, i.e. the absolute sensitivity of the motor. However, the bacterial up-gradient velocity is not simply proportional to the absolute sensitivity, as discussed in the text. In particular, extending the duration of the runs can speed up the velocity as shown in the inset: the running time is reported in seconds on the abscissae while the solid line is Eq (4) with *λ* = 1 and the dashed line is again Eq (4) but *λ* changes now with *τ*_*r*_ so as to maximize the chemotactic velocity. The upshot is that points on the motor curve which do not maximize the absolute sensitivity, like the red one, can actually yield larger up-gradient speeds.

The inset in Fig 1 shows that the term in square brackets grows for *A* = 0 up to *τ*_*r*_ ≲ 3*s*. If *λ* is allowed to vary, the square bracket in Eq (4) has a maximum for *λ* = 2*σ*/(1 − 3*A*). The resulting behavior with respect to *τ*_*r*_ reduces then to the first term in the square brackets discussed previously (see Fig 1). For positive *A*, there is an additional positive contribution to the velocity, which amounts to ≃7% for *A* ≃ 0.03. The inset in Fig 1 shows that extending the running time *τ*_*r*_ as well as having *A* positive, i.e. breaking perfect adaptation, can be advantageous for parameters that are comparable to those of wild-type *E. coli*.

As for the amplitude *K*_0_ in Eq (4), its dependence upon the levels of CheYp is much stronger than for all the other parameters, due to the steep dependence of the motor response shown in Fig 1. Furthermore, the dependence of *K*_0_ on details of the motor response is quite subtle, as discussed below. Linear response theory is again unable to provide a quantitative hold but it can be useful to identify the underlying factors at stake. The linear-regime expression for *K*_0_ reads [30]:

$$K_{0}\propto a\left(1-a\right)\frac{h^{\prime }\left(y\right)\left(1-y\right)}{h(y)(1-h(y))}\;,$$ (5)

where *y* is the fractional concentration of CheYp, *a* is the fractional concentration of CheAp and *h*(*y*) is the clockwise bias motor response in Fig 1.

The expression (5) is proportional to the slope of the curve, i.e. the absolute sensitivity, which is maximum at the inflection point of the motor response. However, the expression (5) contains additional dependencies that reflect the coupling of sensing and running involved in the chemotactic velocity [27]. The effect of those additional factors is that the optimization of the absolute sensitivity does not generally maximize the chemotactic performance [27], as it was also found in [28] by numerical simulations of chemotaxis models [31].

The expression (5) depends on details of the motor response and not just its qualitative sigmoidal shape. For instance, taking a Hill-shaped motor response *h*(*y*) = [1 + (*y*/*y*_0_)^−*H*^]^−1^ and using the quasi-steady state relation *y* = *a*/(*a* + *K*), Eq (5) gives *K*_0_ ∝ *HK*(1 − *a*), which shows that the amplitude is maximal at zero activity *a* = 0. This is intuitively understood by noting that the expression of *K*_0_/(1 − *a*) in the limit of small *y*’s becomes proportional to the relative sensitivity *d* log *h*/*d* log *y*, which is constant for a Hill function. However, an equally sensible allosteric-like shape *h*(*y*) = 1/[1 + *C*((1 + *y*/*K*_1_)/(1 + *y*/*K*_2_))^*n*^] with *K*_1_ > *K*_2_, which is suggested by the conformation spread discussed in [32], gives a maximum amplitude for 0 < *a* < 1/2. This is verified by calculating the derivate of *K*_0_ with respect to *a* at *a* = 0 (where it is positive) and *a* = 1/2 (where it is negative). The two alternatives above subtly differ in their behavior at *y* = 0: the Hill-shaped form vanishes whilst the allosteric one does not (for any finite value of the constant *C*). The non-vanishing is due to the basal finite rate of switching between the two conformations of the motor in allosteric models.

In summary, it is qualitatively clear that maximizing absolute sensitivity, i.e. having the motor set at the point of maximum slope, and perfect adaptation might *a priori* not maximize the chemotactic velocity. However, sharp statements require a detailed knowledge of the bacterial response and its variation along the gradients, which is currently not fully available. For instance, the conclusion [20] based on numerical simulations that imprecision of adaptation has little effect on the rate of chemotactic velocity differs from what will be presented below. Experimental data show that a moderate breaking of perfect adaptation has significant effects in our conditions.

### The chemotaxis response to aspartate is adapted while the response to serine is not

**Fig 2:**
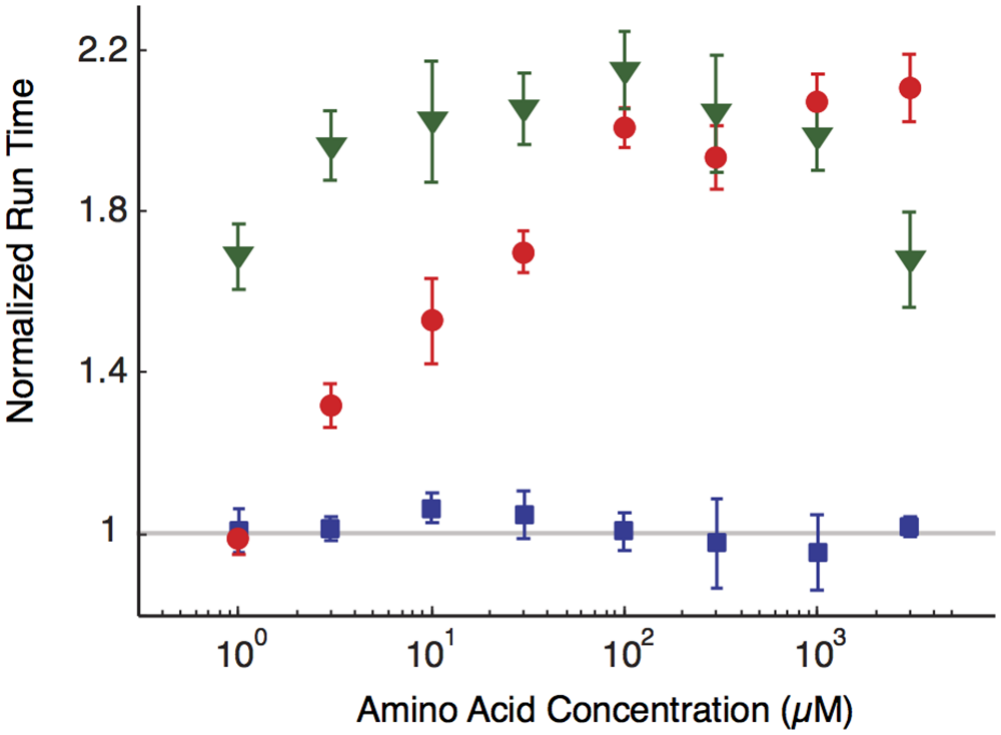
Variation of the bacterial running time with respect to the chemoattractant concentrations. The curves refer to different concentrations of serine (red), aspartate (blue) or aspartate with a background of 30*μ*M of serine (green). Times are normalized to the average running time (whence a non-dimensional quantity on the *y*-axis) in the absence of any chemoattractant, whose average over the bacterial population is ≃1.15s. Run times are calculated by averaging over at least three different experiments and error bars represent the error on the mean. The loss of precise adaptation for the green and the red curves is clearly visible. Note also that the value of the green curve at the lowest aspartate concentration is consistent with the value of the red curve at 30*μ*M, as expected by the fact that the serine background becomes dominant.

We first verified that the adaptation to aspartate and serine for our bacterial strain RP437 (see Materials and Methods) behaves as expected. We measured the mean run times for bacteria in different background concentrations of chemoattractants by standard procedures described in S1 Text. We normalized the run times to the mean run time (∼1.15 s) in the absence of any chemoattractant and reported the normalized values in Fig 2. As expected, the values for aspartate did not change over more than three decades of concentration, whilst the normalized values for serine increased from 1.0 ± 0.1 at 1*μ*M of serine to 2.1 ± 0.2 at 3mM. These behaviors are similar to those for the strain AW405 in Ref. [18].

### Running speed versus the nature and the concentration of chemoattractants

**Fig 3:**
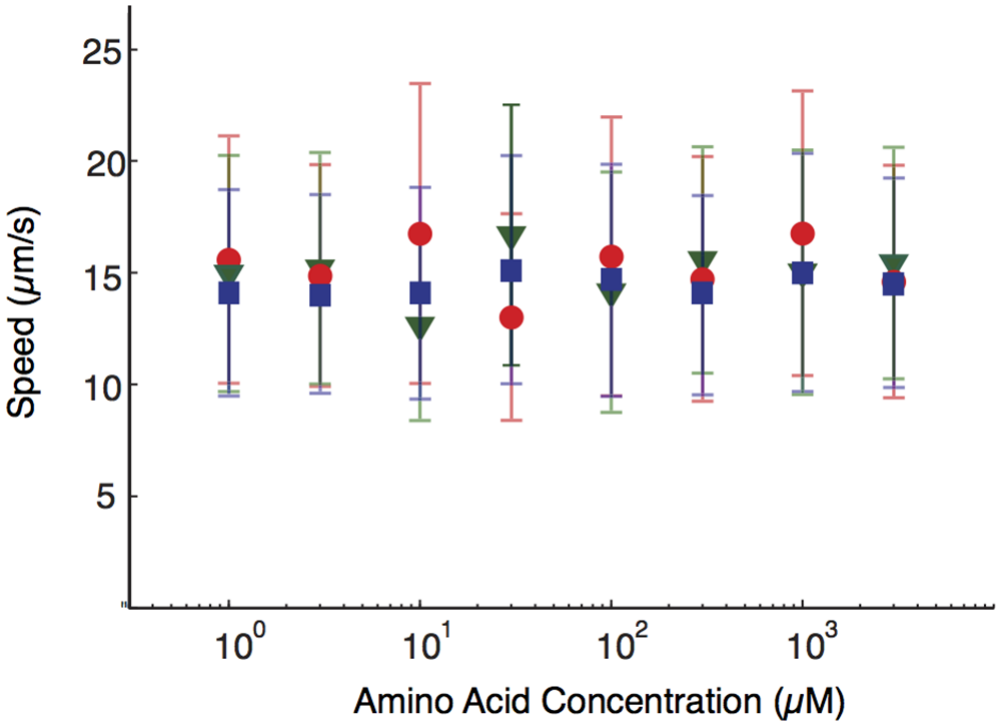
The *E. coli* running speed *vs* the chemoattractant concentrations. As in Fig 2, the three curves refer to serine (red), aspartate (blue) or aspartate with a background of 30*μ*M of serine (green). The mean value is calculated by averaging over the population of bacteria and error bars represent the standard deviation of the velocity distribution over the population.

To quantify the role of the running speeds, we tracked bacteria in homogeneous concentrations of serine or aspartate. We filtered tumbling periods and measured running speeds as described in S1 Text. Fig 3 shows the dependence on concentration: the measured mean running speeds in aspartate and serine are 14.5 ± 5.0 *μ*m/s and 15.0 ± 6.0 *μ*m/s, respectively (mean and standard deviation refer to the velocity distribution over the bacterial population), i.e. their values are within the respective error bars.

Our results are in agreement with previous experiments (see Fig. 3 of Ref. [24]) on our same strain RP437. At temperatures >25°C, a strong increase in the running velocity is observed when a background concentration ≳ 300*μM* of serine is added. However, at the temperature 18 ± 1°C of our experiments, the reported dependence of the running velocity on the concentration of serine is consistent with our results. A larger range of concentrations was explored in Ref. [23] for the *E. coli* strain MTCC 1302. The dependence of the running speed on serine concentration was found to be strong and non-monotonic yet at concentrations higher than those of our experiments; in the range up to mM, the dependence found in Ref. [23] is again consistent with our data. Note that the behavior of the RP437 strain differs from the strain AW405, where a 40% variation of the running speed was reported [18]. We chose the strain RP437 because subsequent analyses are simplified if the running velocity is constant.

### Bacteria advance faster in gradients of serine than aspartate

**Fig 4:**
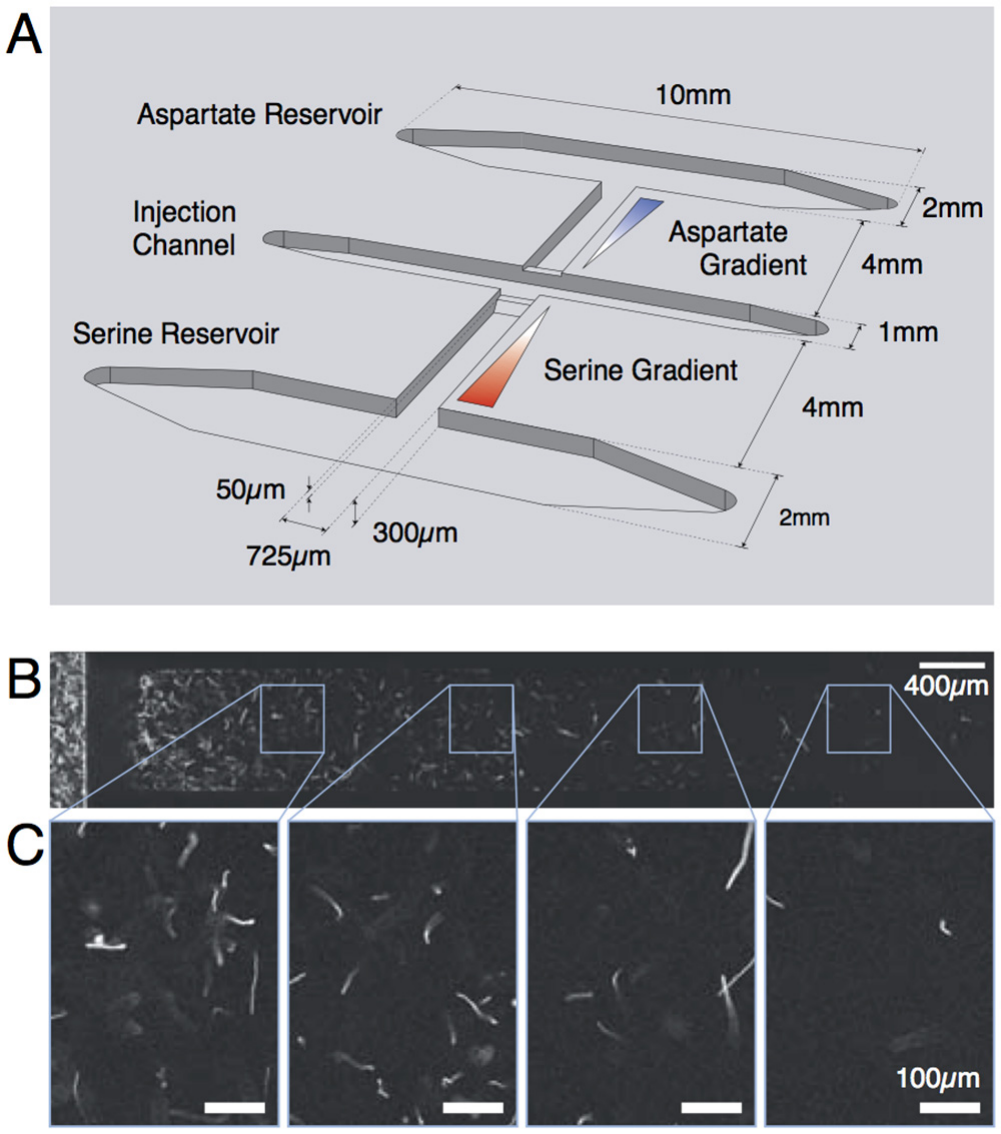
The experimental setup and raw images of bacteria running in the channels. **A.** Illustration of the microfluidic setup where bacterial speed races take place. Reservoirs were filled with the appropriate concentration of chemoattractants and let diffuse through the lateral channels so as to establish linear gradients of chemoattractants in equilibrium with the flow of motility medium applied in the injection channel. Bacteria were then inserted into the injection channel and a fraction of them climb gradients of chemoattractants in the lateral channels. **B.** A typical stitched fluorescence image of a channel. A sequence of 20 images (exposure time of 200ms) were superimposed and then stitched together. On the extreme left, it is shown the injection channels, where the density of bacteria is the highest, while successive positions along the lateral channel are presented moving from the left to the right of the panel. **C.** A zoom of the images in panel B at diverse positions along the lateral channels.

We used the microfluidic device shown in Fig 4 (see Materials and Methods) to measure the progression of *E. coli* in equal-magnitude gradients of aspartate and serine. The main purpose is to show that loss of adaptation to serine does not hamper the climbing of serine gradients, which actually progresses faster than for aspartate. The faster progression reflects the combined effects of different properties of sensitivity and adaptation. The two contributions will be disentangled in the next section by comparing gradients of aspartate with and without a background of serine.

Specifically, the microfluidic device shown in Fig 4 features two different channels with linear gradients of aspartate and serine, respectively. Both gradients range from 0 to 1mM. On the high concentration side, the channels are connected to two reservoirs filled with the corresponding chemoattractant. The reservoirs are large enough that their concentration holds constant throughout the duration of the experiment. On the low concentration side, the channels are connected to the injection channel, where a given bacterial density and lower chemoattractant concentration (here zero) is maintained. Injected bacteria are transported by the hydrodynamic current; a fraction of them spreads into the lateral channels and creates two fronts that climb the corresponding gradients. The bacterial concentration in the injection channel is *OD*_600_ = 0.05, i.e. ∼4 × 10^7^ bacteria per ml. That density empirically guarantees that the number of bacteria in the lateral channels is large enough for reliable statistics yet low enough for convenient imaging and to ensure that the distortion of the gradients due to the bacterial consumption of chemoattractants is negligible (see S1 Text). The reservoirs act as sinks (on the timescale of our experiments) for the bacteria arriving at the end of the channels.

**Fig 5:**
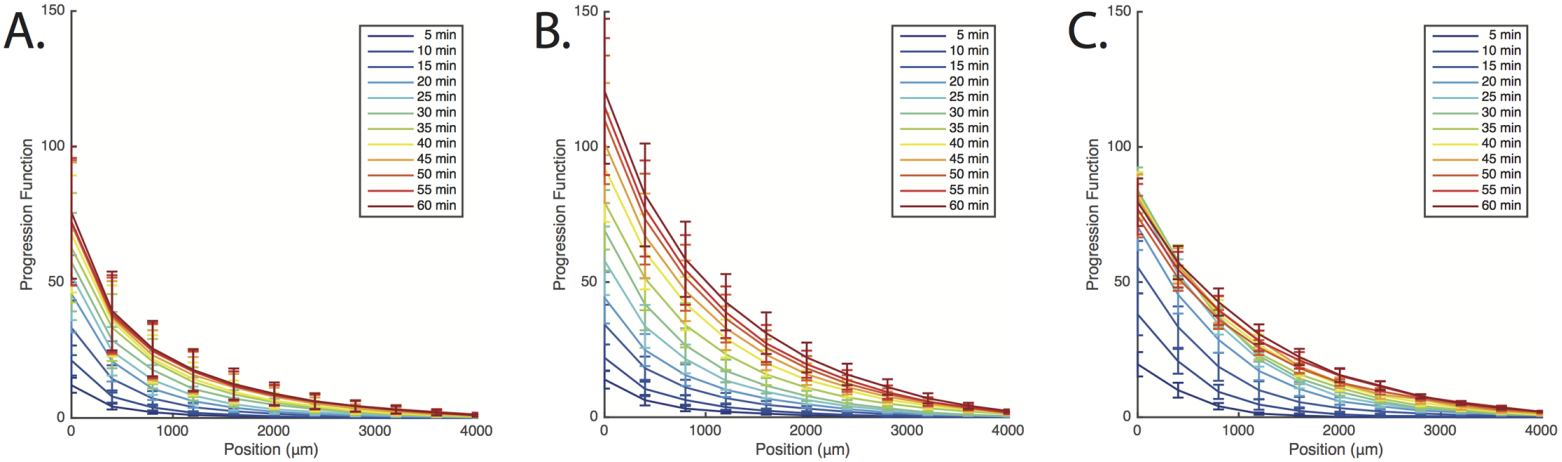
The progression of bacteria in the lateral channels. The graphs show at different times the so-called progression function, i.e. the distribution function of the number of bacteria cumulated from the position indicated on the abscissae up to the end of the channels on the side of the reservoirs. Curves were obtained using five different experiments. Panels A, B and C show the progression function for a gradient of aspartate, of serine, of aspartate with a background of serine, respectively. All the gradients go from 0 (at the entry of the channel) to 1mM.

**Fig 6:**
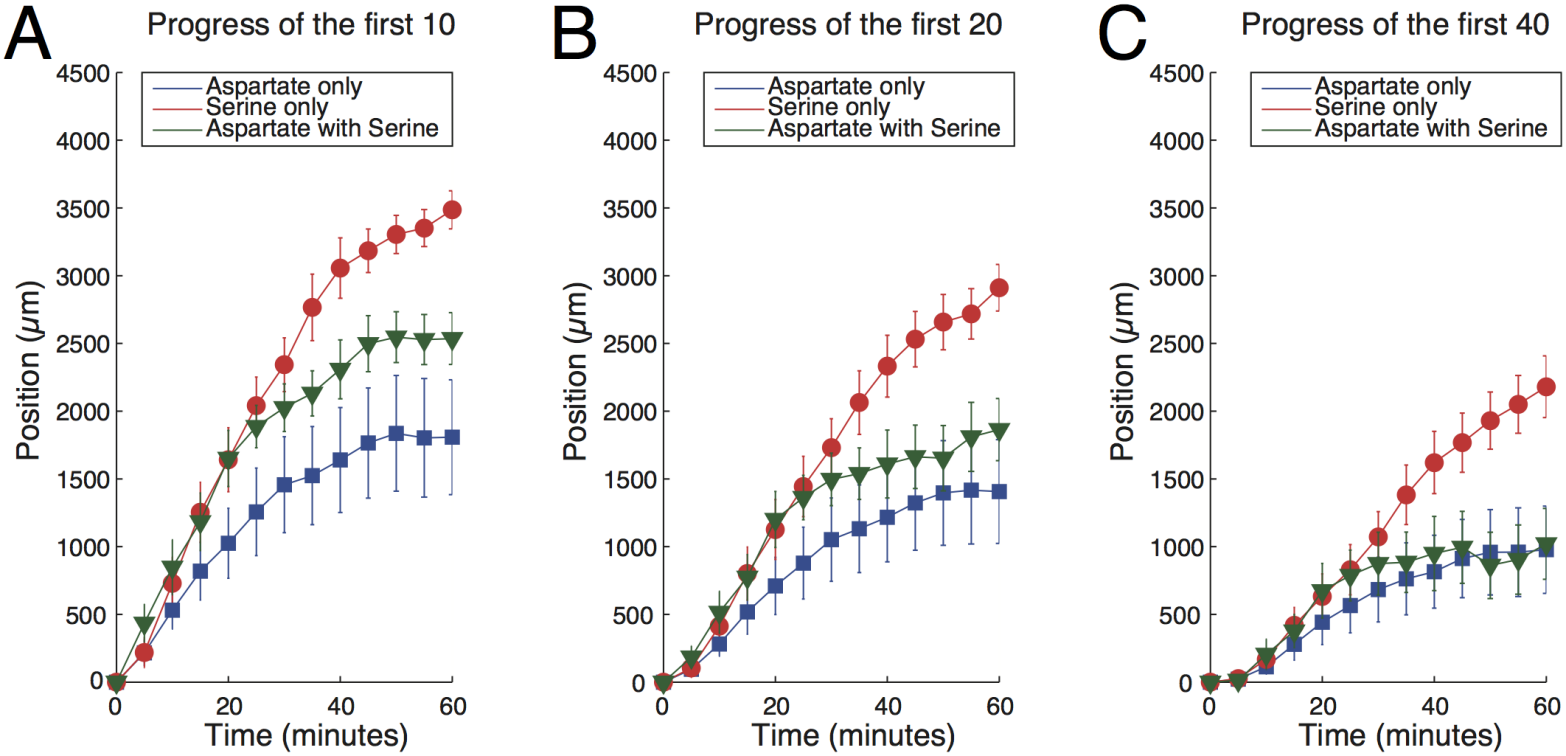
The bacterial forefronts *vs* time. We show the position of the (A) 10^*th*^, (B) 20^*th*^ and (C) 40^*th*^ most advanced bacteria for a gradient of serine (red), a gradient of aspartate (blue) and a gradient of aspartate with a 30*μ*M background of serine (green). Curves represent the mean of five experiments and error bars represent the error on the estimation of the mean.

To quantify the progression of bacteria in the lateral channels, we measured the number of bacteria in the channels as a function of time. In particular, we measured the “progression function”, i.e. the cumulative distribution of the number of bacteria summed from a given location to the end of the channel (on the reservoir’s side). Fig 5 shows that the progression function in the advanced part of the channel raises faster for serine than aspartate. This is also confirmed by extracting the bacterial positions at different times from the images and, for each time, ranking them in increasing order along the channel coordinate (see SI). In Fig 6 we show the position of the 10^*th*^, 20^*th*^ and 40^*th*^ most advanced bacteria. The progression is approximately linear for the first 20–30 minutes, followed by a decrease in slope and eventual saturation, which will be discussed below. For the 10^*th*^ most advanced bacterium in the first 30 minutes, we measured a slope of 1.5 ± 0.2 *μ*m/s for the serine gradient, whereas in the aspartate gradient the slope is 0.85 ± 0.14 *μ*m/s. Similar results are found by considering the 20-th or 40-th bacterium (see Fig 6). Note that the linearity of the progression versus time is only approximate: our linear fit is meant to give an idea of the velocities and their differences and should not be taken as suggestive of a perfectly constant velocity along the gradients. Indeed, even for aspartate, the sensitivity of the response is expected to change along the linear gradient because of the Weber-type response. For serine, loss of adaptation will further lead to an increase of the running time along the gradient. The role of loss of adaptation in the faster progression will be directly assessed in the next section but can already be surmised from the fact that the curves for serine and aspartate start similarly (when the running times are comparable) and their difference gets more pronounced as time elapses (and the respective running times diverge).

The shape of the progression function in Fig 5 is compared to results of numerical simulations in the SI. We also verified that the progression of bacteria is genuinely due to chemotaxis, viz. the control experiment with a constant profile of chemoattractants without any gradients, features a much slower progression (see SI).

In conclusion, loss of precise adaptation does not impair the climbing of gradients of serine, which is actually faster compared to the climbing of aspartate gradients with the same slope and extension. Our *E. coli* strain has of course different sensitivities to aspartate and serine, which is the motivation for the experiments hereafter.

### A uniform serine background disrupts adaptation to aspartate and speeds up bacterial progression in aspartate gradients

We tried inducing loss of precise adaptation to aspartate by adding a constant background of serine. The appealing feature is that we could then directly compare the response to the same chemoattractant with or without adaptation. If loss of precise adaptation indeed leads to a larger chemotactic velocity, we expect that the same aspartate gradient should lead to a stronger progression of bacteria when a serine background is present.

The rationale for expecting loss of precise adaptation to aspartate in the presence of a background of serine goes as follows. Methylation processes are responsible for the feedback that controls the adaptation of the chemoreceptors [4–7]. Loss of precise adaptation is due to the reduced availability of occupation sites for (de-)methylation on the receptor clusters. Serine (Tsr) and aspartate (Tar) preferential receptors jointly participate in the allosteric clusters and assist each other in methylation process [33–37]. Chemotactic responses should then be affected by the presence of multiple signals that are integrated by the chemotaxis pathway. Some experimental evidence supporting this hypothesis was previously reported in Ref. [19], *viz.* the kinase activity of CheA measured by FRET for different combinations of chemoattractants agrees with the predictions of the allosteric models. A prediction of those models is that the more abundant Tsr receptors assist Tar receptors in keeping their adapted state [38]. Therefore, we expect that the addition of serine increases the methylation level of receptor clusters and the ensuing reduction in available sites leads to loss of precise adaptation to aspartate.

We tested the previous prediction by adding a constant background of 30*μ*M of serine and varying the concentration of aspartate within the same range as in Fig 2. We measured the run time of bacteria and found that the mean run time (normalized again to the value without any chemoattractants) increased from 1.7 ± 0.2 at 1*μ*M to 2.1 ± 0.2 at 100*μ*M, showing that adaptation to aspartate is indeed altered by a background of serine. We then verified that the running velocity is weakly affected by the presence of the serine background (see Fig 3). Finally, we performed the speed race assay in the aspartate channel with a background of 30*μ*M serine (see Fig 6) to demonstrate the role of the loss of precise adaptation on the chemotactic velocity. The approximate slope of the bacterial progression indeed increased in comparison to the channel with aspartate only, e.g. the slope for the most advanced 10^*th*^ bacteria increased from the value 0.85 ± 0.14 *μ*m/s previously reported, to 1.31 ± 0.07 *μ*m/s.

### Saturation

The progression of the 10^*th*^, 20^*th*^ and 40^*th*^ most advanced bacteria slows down at long times for all attractants, irrespective of adaptation, and eventually saturates (see Fig 6). Saturation sets in when the curves in Fig 5 approach a steady profile and saturation levels depend on the choice 10, 20, 40. These observations suggest the following specific mechanism, in addition to the general remark that the relative concentration gradient ∇*c*/*c* decreases along linear profiles.

When bacteria reach the end of the lateral channels and penetrate into the reservoirs, they disappear from our images. Due to the size of the reservoirs, this is equivalent to an absorbing boundary condition at the end of the channels. During the phase when all bacteria are advancing in the lateral channels, the frame that contains the most advanced individuals (10,20,40) systematically progresses with them. However, when advanced bacteria start to be absorbed in the reservoirs, the frame containing the most advanced individuals shifts backward to total again 10, 20 or 40. Bacteria in the channel still move forward yet the backward shifts of the frame and the inclusion of less advanced bacteria entail a slow down of the progression curve in Fig 6. When influx and outflux of bacteria eventually balance, the density in the lateral channels reaches a stationary profile and the progression function becomes constant.

The intuitive arguments above are supported by numerical simulations of the drift-diffusion equation derived in Ref. [27]. We take a diffusion constant consistent with the run time and the velocity in Figs 2 and 3 and parameters of the drift consistent with those measured in Ref. [39]. The resulting progressions, profiles and timescales of saturation are compatible with our experimental observations. Since the saturation effect is not directly related to the role of loss of adaptation, we refer to the S1 Text for details.

## Discussion

Our experiments show that a moderate loss of precise adaptation does not impair the climbing of serine gradients, which is actually faster than for equal-magnitude gradients of aspartate over the same, extended range of concentrations. The comparison between gradients of aspartate in the presence/absence of a background of serine directly demonstrates the role of the loss of precise adaptation. We showed that the sped-up progression in the channels largely results from the increase of the run time from its value ≃1.15s in the absence of any chemoattractant. Since the previous value of the run time is not peculiar to our *E. coli* strain RP437 and media, we expect that similar results hold for other conditions and strains.

What are the functional and evolutionary implications of our results? An important *caveat* is that quantitative assessments of the selective advantages brought by chemotaxis and its ecological conditions of selection are essentially unknown. The common sense in the field is that selective pressure on chemotaxis is important and the drive toward effective chemotactic performance is substantial, which is the point of view pursued hereafter. However, we stress that the level of differences in the chemotaxis performance that are significant for evolutionary selection is an important open issue.

Our results suggest that precise adaptation to aspartate is not due to the need of efficient climbing of static and extended gradients. If that were the main functional pressure, a moderate loss of precise adaptation would be preferable, as shown by our experiments adding a background of serine. Actually, there is no physiological support to consider the response to aspartate as paradigmatic and the response to serine as accidentally imperfect. For instance, ring-forming assays, which combine growth and motility, show that the serine-chasing ring of bacteria is the first to spread from an initial inoculum, later followed by the aspartate ring [21]. A similar observation is made in capillary assays [40]. Furthermore, *E. coli* chemotaxis toward amino acids correlates with their utilization [41]. Serine is consumed earlier than other amino acids in tryptone broth [42] and reduces growth at high concentrations [20, 43–45]. Based on these facts, it is unlikely that *E. coli* selective pressure on the response to aspartate is stronger than to serine.

More generally, we find it unlikely that chemotaxis is selected for climbing simple profiles, like constant linear and exponential gradients usually considered in the laboratory. In Ref. [27] we raised the possibility that physiological conditions might be complex, with gradients strongly varying and fluctuating. A reason mentioned in [27] is the uptake of chemoattractants by bacteria in the colonies that they form as they grow, coupled with the scarce levels of attractants in the conditions where chemotaxis is likely to be important. Strongly varying gradients were also recently inferred for *E. coli* in Ref. [46] and constitute the typical environment for marine bacteria [47]. The important point in fluctuating profiles is that chemotaxis does not involve climbing of gradients only, yet also maintaining contact with the peaks of the profile. As we have shown here, climbing of gradients is favored by a non-adapted response with a running time that increases with concentration. At the level of the impulse linear response, that corresponds to a positive lobe stronger than the negative one. The task of maintaining contact with peaks of the chemoattractant profiles was analyzed theoretically in Ref. [26] and numerically in Ref. [20]. They both show that the optimal linear response is not adapted and should feature an impulse response with a strong negative lobe. The optimal degree of adaptation in fluctuating profiles should then involve a trade-off between the positive and the negative lobes in the impulse response, which depends on the environmental conditions and might result in perfect adaptation if fluctuations are sufficiently strong [27]. Future experiments with controlled fluctuating environments will be needed to test those predictions.

A final conjecture is that the degree of adaptation to aspartate or serine might actually depend on environmental conditions. Allosteric models for chemotaxis predict that the degree of adaptation is controlled by the relative levels of Tar and Tsr receptors [33–38]. Furthermore, the levels of the two types of receptors change with the state of the bacterial colony [48, 49]. It is then likely that the relative predominance of Tsr over Tar receptors changes, e.g. with the conditions of culture and growth. The variations of Tar and Tsr expression levels with the environmental conditions might then provide informative clues on bacterial chemotaxis.

We conclude stressing that the function of biological systems is a notoriously tricky issue, yet it is essential to understand what molecular pathways are doing, what is evolutionarily shaping them and to go beyond the list of their parts. Chemotaxis has been thoroughly investigated and we have a unique knowledge of the pathway and its molecular components [5]. It is thanks to this knowledge that one can concretely ask functional questions and they seem to call for a stronger coupling with bacterial metabolism and physiology.

## Materials and Methods

### Bacterial strain and growth

For the study reported here, we used the RP437 strain. We electroplated the pBRBRO plasmid, a colE1-based plasmid bearing the mOrange gene under the control of a leaky promoter (Tac) considering no tight repressor allele (*lacI*^*q*^) were present in neither the strain nor the plasmid. Single colonies were picked from a fresh plate and were grown overnight in Tryptone Broth (TB) supplemented with the appropriate antibiotics. The saturated culture was pelleted and resuspended in the same volume of TB. The washed culture was diluted to OD = 0.002 and allowed to grow up to OD = 0.2 − 0.3. Cells were harvested and washed 3 times in the motility medium [18] prior to injection in the microfluidic setup. They were then diluted to have an OD = 0.05, which corresponds to ∼4.10^7^ bacteria/ml.

### Microfluidic setup

The channels were carved into a plastic piece (PMMA) using a micromilling machine (MiniMill/GX, Minitech Machinery) and appropriate carbide tools (NS tool). Inputs and outputs to the channels were pierced using a 600*μ*m drill (Performance Micro Tool). The carved channels were closed with a glass coverslip using a UV glue (NBA107, Norlands). The assembled setup consists of two reservoirs (10 x 2 x 0.3 mm) connected to an injection channel (10 x 1 x 0.3 mm) through the lateral channels (4 x 0.725 x 0.3 mm) where bacteria climb the gradients. At the junctions between the channels, ridges of 250/300*μ*m in height/length were added to reduce the extension of the flow from the injection channel.

### Procedure

The reservoirs were filled with a 1mM solution of the appropriate amino acid together with fluorescein that has roughly the same diffusion coefficient as the chemoattractants. The input and output of the reservoirs were sealed using adjusted metallic plugs. A flow of the same motility buffer without chemoattractant was then applied in the injection channel. A linear stable gradient took about 3 hours to form, as checked by imaging the fluorescein. The input of the injection channel was then switched to a solution of bacteria. The flow was maintained using a syringe pump at 20 *μ*l min^−1^. Flow was interrupted during acquisition of images of the channels, which were taken every 5 minutes. Images of bacteria and their density in the channels were extracted using the automated image analysis program Fiji [50] (see S1 Text for further details).

## Supporting Information

S1 Text Contains extended experimental procedures, namely to quantify the bacterial progression, estimates of consumption effects in our experiments, modeling of the bacterial motion and theoretical expressions of the chemotactic velocity. (PDF)
