# Supplementary material for: The Role of Adaptation in Bacterial Speed Races
Source: PLoS Comput Biol. 2016 Jun 3;12(6):e1004974. doi: 10.1371/journal.pcbi.1004974 (PMC4892596; doi:10.1371/journal.pcbi.1004974)
Supplement: S1 Text — (PDF) [file pcbi.1004974.s001.pdf]

Supplemental Information to "The role of adaptation in bacterial speed races" by J. Wong-Ng et al.

1 Extended experimental procedures

1.1 Measuring swimming speeds and mean run times

To determine the properties of bacteria reported in Figs. 1 and 2 of the main text, we proceeded as follows.

Bacteria prepared as described in the Materials and Methods section of the main text, were injected in a 200μm-deep microfluidic chamber. The concentration of chemoattractants in the chamber was kept constant in space and time. After imaging bacteria in phase contrast, trajectories were extracted using the algorithms and the procedure that we developed and exploited in previous work [1]. First, we subtracted the background image. Then, an edge filter algorithm was applied to single out bacteria. Smoothing of the images gave maximum-centered objects and the position of the bacteria was assimilated to those local maxima above a threshold, which was fixed by experimentation. Only trajectories of more than 5 seconds were kept to enhance reproducibility. Finally, the mean run time for a given condition was estimated as the cumulated time spent running (vs tumbling) for all the trajectories, divided by the number of switching events from run to tumble in the whole population. Running/tumbling events were identified by the same procedure that we employed and described in detail in [1]. Swimming speeds were calculated for each trajectory by taking the difference between successive positions of the bacteria. We discarded time intervals corresponding to tumblings with an additional extension of 0.2s on both sides of each interval to avoid biases due to the slow-down of the velocity associated to tumbling events.

1.2 Extracting bacterial densities in race channels

The lateral channels of the microfluidic device described in the main text feature the speed races performed by bacteria. The density of bacteria in the channels was obtained from fluorescence images and the progression of the fronts as follows.

For each time step and each position in the channel, 20 fluorescence images were acquired with an exposure time of 0.2 s. After subtraction of the background and smoothing, the variation in the fluorescence intensity over noise ($\Delta F/F$) corresponding to the bacteria was systematically over 1, as shown in Fig. S1. The bacteria could then be assimilated to moving objects and identified by keeping only the local maxima with $\Delta F/F > const$. The constant was fixed by experimentation to the value 5 and the filtering was implemented by a standard process in the Fiji plugin [2]. Note that images are taken only in the lateral channels, i.e. bacteria that reach the reservoirs are not imaged. The relatively large size of the reservoirs results in their acting as bacterial sinks (at least for the typical time of the experiments reported in this work).

To further minimize effects due to fluctuations in the detection, the object extraction method that we just described was performed on the ensemble of the 20 images for each position and time step. The average of the objects per image was the

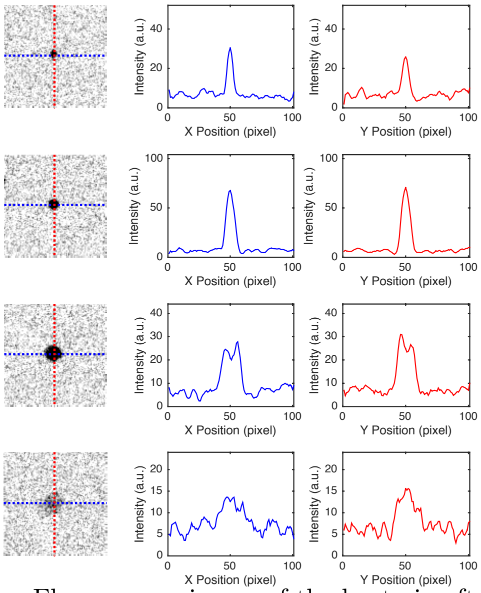

Figure S1. Left column : Fluorescence image of the bacteria after background subtraction, Gaussian blur and LUT inversion. Middle and right columns : Fluorescence intensity along the x axis (blue dashed lines) and the y axis (red dashed lines) of the left column. Each row represents different examples of detected objects.

proxy that we employed for the number of bacteria at that position. From the number of bacteria at each position, we reconstructed the bacterial density distribution and the progression function, that is the cumulative distribution of the number of bacteria summed from a given location until the end of the channel (on the reservoir's side). The above procedure yielded the curves shown in Fig. 4 of the main text.

Finally, to prevent the effect of missing some bacteria, we constructed the progression function and extracted the position of the 10th, 20th and 40th most advanced bacteria by linear interpolation. While this method entails a smoothing of the data, it is more robust to problems in annotation and imaging than the alternative method of considering the position of each bacterium. We tested our method on synthetic data generated by simulations and verified that missing a few bacteria in the conditions of our experiments leaves the results of our procedure essentially unmodified.

2 Bacterial progression in a background concentration of aspartate or serine

In order to verify that the progression within the channels reported in the main text is germane to chemotaxis, we submitted the bacteria to a constant level (100μM) of serine or aspartate. These controls (see Figure S2) show that the progression due to diffusion is indeed much slower (roughly one order of magnitude) than the progressions obtained in the presence of gradients. The square-root diffusive behavior is obviously not negligible with respect to the linear-in-time drift at very short times yet this is limited to the very first few minutes. We verified that including or leaving aside those initial few minutes plays a negligible role in the estimation of the drift velocity. It is also worth noting that constant backgrounds of aspartate or serine have comparable rates of progression.

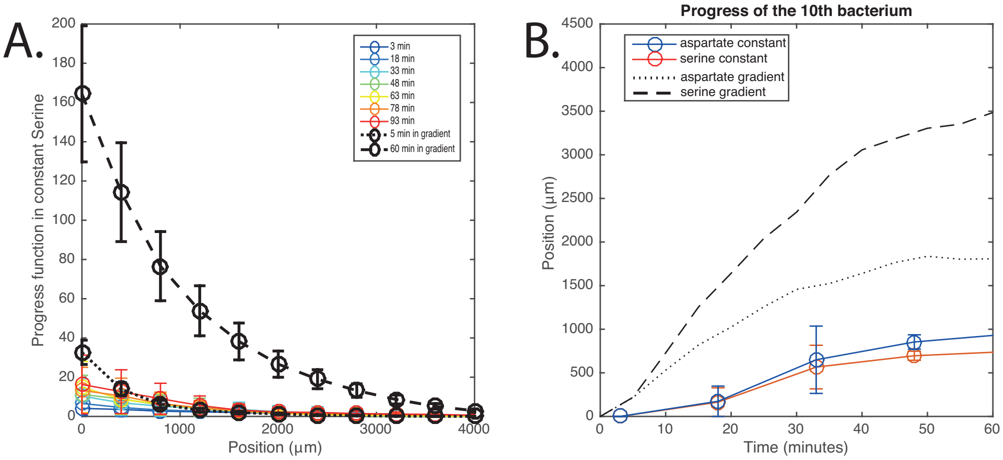

Figure S2. Control experiments with constant backgrounds of aspartate or serine. A. The progression function of the bacteria in a constant background of serine. The progression functions for a gradient of serine at 5 and 60 minutes were added for comparison. B. Progression of the 10th bacterium in a constant background of aspartate (blue) or serine (red). The curves of the progress in gradients of aspartate and serine reported in the main text were added for comparison.

3 Consumption effects are negligible for our experiments

The goal of this Section is to provide estimates for the consumption of chemoattractants by the bacteria in our experimental conditions. The analysis shows that the linear attractant profiles established in the lateral channels before the injection of bacteria, are not significantly altered during the experimental speed races.

The uptake rate of aspartate is ≈ 10 nmol/min/(mg of cell protein) [3]. Using that *E. coli* has a cell protein content of ≈ 0.2pg per cell (see page 33 in [4]), it follows that the uptake rate is $\alpha \approx 20,000$ aspartate molecules per second per cell. The cell number density in the injection channel of our microfluidic setup is $n \approx 4 \cdot 10^7$ cells/ml and the number of bacteria in the lateral channels (where speed races actually take place) is at least three orders of magnitude smaller. The latter estimate is obtained noting that few hundred bacteria are present in the entire lateral channels even at the latest times of our experiments (see Fig. 4 in the main text and Fig. S3 below). The estimation is conservative as the density of bacteria is actually lower at the edge than in the back of the progressing fronts (see again Fig. 4 in the main text and Fig. S3 below). By using the uptake rate α above and the value of n for the injection channel, we obtain that the rate of consumption $\alpha n \approx 1$ nM/s. Dividing the rate of consumption by the velocity of drift $v \simeq 1\mu m$/s that we measured in our set-up (see main text), we obtain for the gradient created by consumption ≈ 1 nM/μm. This value is already more than two orders of magnitude smaller than the gradient 0.25 mM/mm that we maintain in the lateral channels. Moreover, the consumption rate in the lateral channels is at least three orders of magnitude smaller, as discussed above. We conclude that the aspartate linear profiles in the lateral channels where speed races take place, are unaffected by bacterial consumption.

Consumption rates of serine have the same order of magnitude as those for aspartate (see Fig. 2 in [5]) ; the same conclusions drawn above for aspartate apply then to serine.

4 Modeling of the bacterial up-gradient drift in the channels

Fig. 5 in the main text illustrates the progression of the 10th, 20th and 40th most advanced bacteria vs time. For short times the progression is roughly linear whilst the fronts in the lateral channels slow down at later times ($t > 20$min) and the progression function saturates. The purpose of this section is to give further details on the saturation process and the mechanism intuitively explained in the main text.

We take images in the lateral channels only, as discussed in the main text and above. It follows that those bacteria which penetrate into the reservoirs (where a constant level of chemoattractant is maintained) stop being processed. This is equivalent to having an absorbing boundary condition at the end of the channels. As long as all bacteria are advancing in the lateral channel, the frame containing the most advanced individuals (10, 20 or 40) systematically progresses with them. However, when bacteria start reaching the end of the channels, the frame containing the most advanced individuals shifts backward to include additional bacteria and number again 10, 20 or 40. When the in- and out-fluxes in the lateral channels eventually equilibrate, a steady state is achieved and there is no net advancement of the frames. This is the intuition for the mechanism contributing to the observed saturation of the progression function. This mechanism and the general dependence of the chemotactic response on the relative concentration gradient $\nabla c/c$, capture the main effects of saturation as we proceed to show.

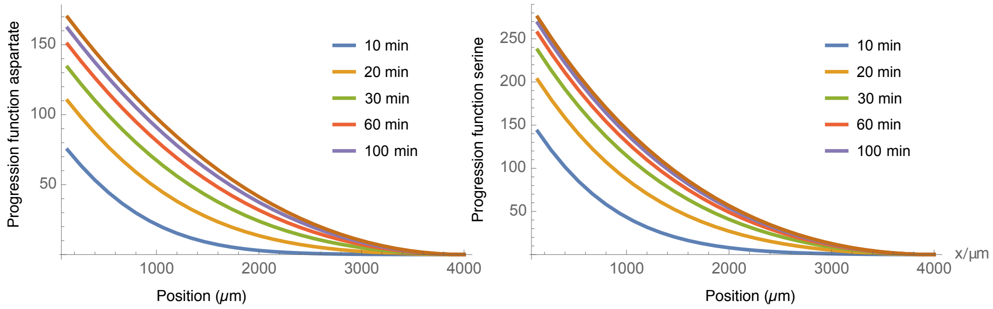

Figure S3. The progression function for numerical simulations of the model Eq. (1) for the dynamics of bacteria in aspartate (left) and serine (right) channels of length $L = 4$mm. The choice of the parameters of the model is discussed and motivated in the text. The progression function is defined as the cumulative distribution of the number of bacteria summed from a given location (shown on the abscissae in μm) to the end of the channel, where an absorbing boundary condition is imposed. The absorbing boundary reflects the fact that we experimentally take images only in the lateral channels and the large size of the reservoirs. Note that the slope of the curves decreases with x, i.e. bacterial density is higher in the back than at the edge of the fronts.

We performed numerical simulations of the following partial differential equation describing the bacterial concentration $n(x,t)$ in a one-dimensional chemoattractant profile $c(x)$ (see [6] for details):

$$\partial_t n = -\nabla\left(\chi n \nabla c\right) + D_0 \nabla^2 \left(1 + \gamma c\right) n. \qquad (1)$$

Here, D_0 is the effective diffusion coefficient, which accounts for the random component of the run-and-tumble motion. Its order of magnitude is $v^2 t_r$, where v is the bacterial speed and t_r is the run time. The parameters χ and γ in Eq. (1) account for the response of the chemotactic pathway to chemical stimuli. In particular, χ is the

chemotactic coefficient mentioned also in the main text. The parameter γ relates to the level of adaptation: the response is perfectly adapted for $\gamma = 0$ while increasing values of γ correspond to loss of precise adaptation, namely a positive lobe more pronounced than the negative one in the impulse response function [1, 7].

To determine the parameters in Eq. (1), we combined known results on the chemotactic pathway with our measurements of the bacterial run time and speed. The diffusion constant was set to $D_0 = 10^3 \mu m^2/s$ both for aspartate and serine channels. We used $\gamma_{\rm asp} = 0$ for bacteria in the aspartate channel whilst for serine we chose a value to account for the experimentally measured increase in the run time. Specifically, since the run time doubles as the serine concentration changes from $10\mu M$ to $10^3\mu M$ (cf. Fig. 1 in the main text), we took $\gamma_{\rm ser} = 0.001\mu M^{-1}$. For the chemotactic coefficient χ, we assumed the Weber's law scaling $\propto \nabla c/c$, which translates into $\chi \propto 1/x$ for our experimental linear gradient. The constant was adjusted to match the velocity of the fronts presented in the main text. We finally obtained $\chi_{\rm asp}\nabla c = \frac{0.85}{x}\mu m^3/s$ and $\chi_{\rm ser}\nabla c = \frac{1.5}{x}\mu m^2/s$, having used the value of the slope $\nabla c \simeq 0.25\mu M/\mu m$ in our experimental set-up.

We simulated Eq. (1) for a channel of length $L = 4mm$, as in our experimental setup. At the right boundary (on the side of the reservoirs) we imposed an absorbing boundary condition, i.e. the bacterial density drops to zero, $n(L) = 0$. At the left boundary (on the side of the injection channel) we modeled the bacterial influx by an effective reservoir with fixed density ρ, which we use as a fitting parameter. The values $\rho_{\rm asp} = 0.1$ for aspartate and $\rho_{\rm ser} = 0.2$ for serine yield total numbers of bacteria in the respective channels that are consistent with the ones observed experimentally (compare Fig. S3 and Fig. 4 in the main text). The order $\rho_{\rm ser} > \rho_{\rm asp}$ is consistent with the fact that bacteria entering the lateral channels already sense the chemoattractant gradients and with the higher affinity for serine than aspartate that was reported in the main text.

The progression function obtained by numerical simulations of the model Eq. (1) compares well with the one measured in the experiments, see Fig. S3. The position of the most advanced 10th, 20th and 40th bacteria is then extracted from the cumulative distributions in Fig. S3 and shown in Fig. S4. The leveling off of the curves is clearly visible and accentuates as the cumulative distributions reach their steady-state profiles in Fig. S3. Time and length scales of the saturation are consistent with the experimental findings reported in the main text. We conclude that the leveling off of the progression curves is well captured by the simple physical ingredients introduced into Eq. (1).

We also checked that our results hold when additional effects are included, e.g. receptors saturate at high concentrations and Weber's law is then violated. To have a sense of the importance of those effects, it is convenient to use the phenomenological approach proposed in [8]. Specifically, the concentration c is replaced by a function $\phi(c)$, which leads to replacing Eq. (1) by

$$\partial_t n = -\nabla\left(\xi n \nabla\phi(c)\right) + D_0\nabla^2\left(1+\gamma\phi(c)\right) n \,. \tag{2}$$

The general function ϕ is chosen to ensure that Weber's law obtains at concentrations below the saturation of receptors and a faster decay ($\propto \nabla c/c^2$ in standard allosteric models, see e.g. [9]) is obtained beyond saturation. A convenient functional form that respects those desiderata is:

$$\phi(c) = \alpha \ln\left[\frac{1+c/K}{1+c/K'}\right] , \tag{3}$$

which indeed gives Weber's law for $K \lesssim c \lesssim K'$ and crosses over to $\propto \nabla c/c^2$ for $c \gtrsim K'$. The constant α in Eq. (3) is adjusted empirically, while ξ in Eq. (2) is fixed by the match the expression of χ in Eq. (1) for the Weber's regime. The binding constants measured in [10] for aspartate are $K = 0.2\mu M$ and $K' = 30\mu M$. Results of numerical

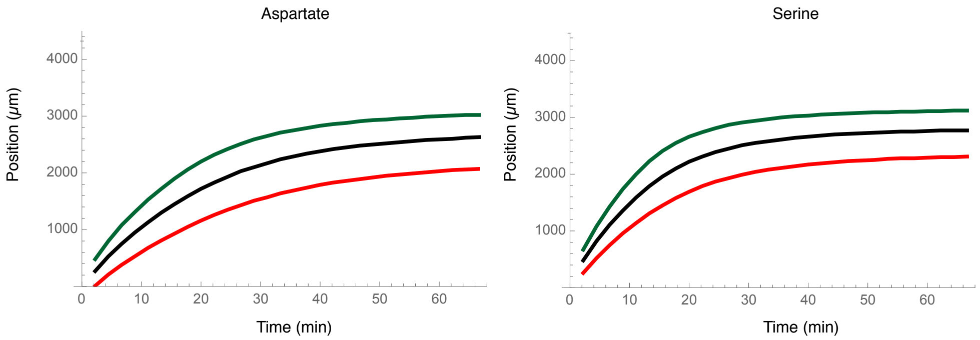

Figure S4. The progression of bacterial forefronts vs time for numerical simulations of the aspartate (left) and the serine (right) channels. The curves in green, black and red correspond to the progression of the 10th, 20th and 40th most advanced bacteria. The choice of parameters in the model Eq. (1) is discussed and motivated in the text. Note the leveling off of the curves as time progresses, which reflects the steady state established in the channel by the balance of injection and absorption of bacteria (on the left and the right, respectively).

simulations of the model Eq. (2) (see Fig. S5) are comparable to the ones obtained above with the original model Eq. (1) suggesting that additional terms included into Eq. (2) have moderate effects.

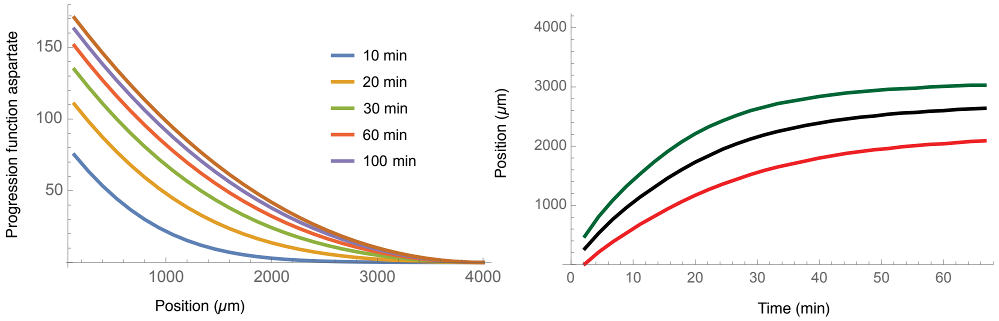

Figure S5. Numerical simulations of the model Eq. (2), which includes a phenomenological account of receptors' saturation. The progression function is shown in the left panel for the aspartate channel; the corresponding bacterial forefronts vs time are shown in the right panel. Green, black and red curves correspond to the 10th, 20th and 40th most advanced bacteria. The choice of the parameters of the model is discussed in the text and the nonlinear function ϕ is given by Eq. (3).

5 Theoretical expressions for the chemotactic velocity

In this section we present the results of numerical simulations of bacterial run-tumble motion in a linear concentration profile with a non-adaptive response. We compare the results with the linear response theory – eq. (1) of the main text.

Tumbles reorient the direction of motion by a random angle φ with a given

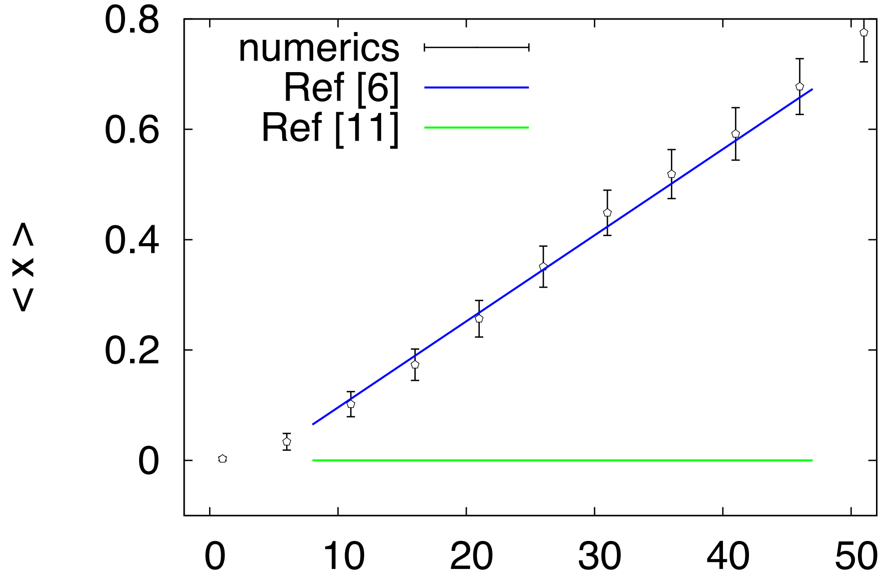

Figure S6. The average position of a group of bacteria initially positioned at the origin of a linear gradient profile of chemoattractant. The curve shows the average position $\langle x \rangle$ in the direction of the gradient as a function of time. The parameters of the dynamics, as defined in the text, are $\langle \cos\phi \rangle = 1/2$, $u = 20\,\mu$m/s, $\tau_0 = 1$ s, $\lambda = 0.5$ s^{-1}, $K_0 = 5 \cdot 10^{-5} \mu$M^{-1} s^{-1}, $g = 1\mu$M$/\mu$m, $c_0 = 1$ mM.

distribution and they occur according to a inhomogeneous Poisson process with rate

$$\frac{1}{\tau_r} = \frac{1 - Q(t)}{\tau_0}\,, \tag{4}$$

where the modulation factor $Q(t)$ is controlled by the chemotactic response function $K(t)$ as

$$Q(t) = \int_{-\infty}^{t} K(t-s)c(s)ds\,, \tag{5}$$

and τ_0 is the mean run time in the absence of attractant ($c = 0$). The response function that we specifically consider is

$$K(t) = K_0 e^{-\lambda t}\,, \tag{6}$$

which has a unique positive lobe and is therefore manifestly non-adapted. While the arguments in [6] were developed for the general case, we assume here for simplicity that tumbles have zero duration. The chemoattractant concentration profile is assumed linear: $c(x) = c_0 + gx$ and bacteria all start at the origin at the initial time.

Setting $D_{rot} = 0$ (again for simplicity only), one has $\alpha = \sigma$ (see eqs (1) and (2) of the main text) and the chemotactic velocity reads

$$v_d = \chi g = \frac{u^2 \tau_r}{3(1 - \langle \cos\varphi \rangle)} \times \frac{K_0}{\lambda + \frac{1 - \langle \cos\varphi \rangle}{\tau_r}}\,, \tag{7}$$

where $\tau_r \approx \tau_0/(1 - K_0 c_0/\lambda)$.

We simulated the stochastic dynamics described above and report in Figure S6 the average position for 10^7 bacteria of the x component along the direction of the gradient. Results agree with the prediction (7) above (i.e. eq (1) of the main text and Ref. [6]). Conversely, for the current choice of parameters and more generally when $\lambda\tau_0 = \langle \cos\varphi \rangle$, Ref. [11] predicts a zero chemotactic velocity – see eqs. (40) and (41) therein – which differs from numerical results in Fig. S6.

References

1. Masson JB, Voisinne G, Wong-Ng J, Celani A, Vergassola M (2012) Noninvasive inference of the molecular chemotactic response using bacterial trajectories. Proc Natl Acad Sci USA 109:1802-1807.

2. Schindelin J, et al. (2012) Fiji: an open-source platform for biological-image analysis. Nature Methods 9:676-682.

3. Schellenberg GD, Furlong, CE (1977) Resolution of the multiplicity of the glutamate and aspartate transport systems of Escherichia coli. J. Biol. Chem 252:9055-9064.

4. Phillips R, Kondev J, Theriot J (2009) Physical Biology of the Cell. Garland Science, New York.

5. Ogawa W, Kayahara T, Tsuda M, Mizushima T, Tsuchiya T (1997) Isolation and Characterization of an Escherichia coli Mutant Lacking the Major Serine Transporter, and Cloning of a Serine Transporter Gene. J Biochem 122:1241-1245.

6. Celani A, Vergassola M (2010) Bacterial strategies for chemotaxis response. Proc Natl Acad Sci USA 107:1391-1396.

7. Segall JE, Block SM, Berg HC (1986) Temporal comparisons in bacterial chemotaxis. Proc Natl Acad Sci USA 83:8987-8991.

8. Xue C, Othmer HG (2009) Multiscale models of the taxis-driven patterning in bacterial populations. SIAM J Appl Math 70(1):133-169.

9. Celani A, Shimizu TS, Vergassola M (2011) Functional and molecular aspects of bacterial chemotaxis. J. Stat. Phys., 144:219-240, 2011.

10. Vaknin A, Berg HC (2007) Physical response of bacterial chemoreceptors. Mol Biol 366(5):1416-1423.

11. Locsei JT (2007) Persistence of direction increases the drift velocity of run and tumble chemotaxis. J Math Biol 55:41-60.
